# Supplementary material for: HIV-1 DNA sequence diversity and evolution during acute subtype C infection
Source: Nat Commun. 2019 Jun 21;10:2737. doi: 10.1038/s41467-019-10659-2 (PMC6588551; doi:10.1038/s41467-019-10659-2)
Supplement: Supplementary file 1 — Supplementary Information [file 41467_2019_10659_MOESM1_ESM.pdf]

Supplementary Figure 1.

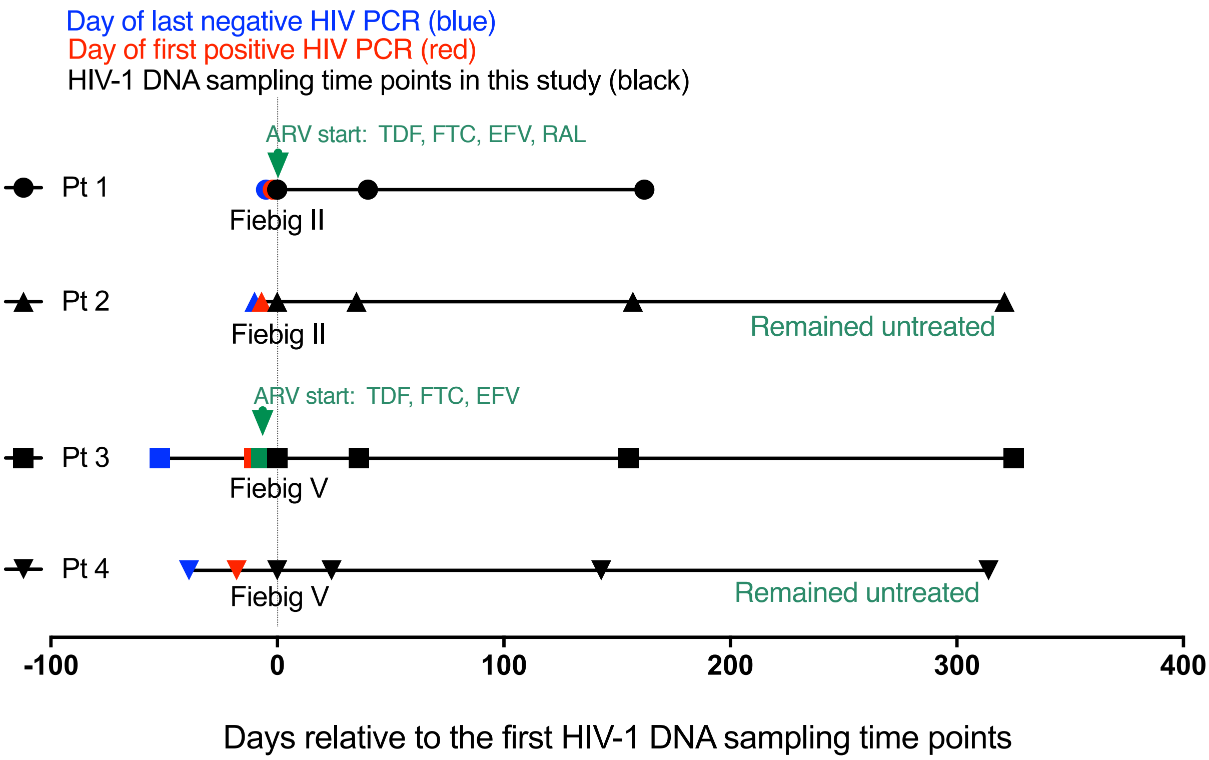

Supplementary Figure 1. Chronological analysis of PBMC sampling timepoint in the study participants (n=4). Each point on the horizontal time lines represents one event: Day of last HIV negative PCR test (blue), Day of first HIV PCR positive test (red), antiretroviral (ARV) treatment start date and first regimen (green). Black points represent the date of collection of PBMC samples examined in this study: The earliest samples for each patient were at stage II, II, V and V respectively.

Supplementary Table 1. Laboratory test results of the study patients.

PATID	Days post last PCR negative test	Days post first PCR positive test	Days post treatment initiation	Viral Load (copies /mL)	CD4 (cells/ mm^3)	Uni-Gold[a]	Determine[b]	Cobas-combi-ELISA	p24-antigen[c]	Centaur -combi-ELISA[d]	Western Blot (Any)	Western blot bands									stage
												p31	gp160	gp120	p65	p55	gp41	p40	p24wb	p18	
Patient 1	5	2	0	3900	851	-	-	-	+	NA	ID	-	-	-	-	-	+	-	-	-	II
		42	40	20	667	NA	NA	NA	NA	NA	-	-	-	-	-	-	-	-	-	-	
		164	162	20	658	-	-	+	NA	NA	-	-	-	-	-	-	-	-	-	-	
Patient 2	10	7	no treatment	380000	457	-	-	+	+	-	-	-	-	-	-	-	-	-	-	-	II
		42	no treatment	37000	596	NA	NA	NA	NA	NA	+	-	+	-	-	+	-	+	+	-	
		164	no treatment	2600	448	NA	NA	NA	NA	NA	NA	NA	NA	NA	NA	NA	NA	NA	NA	NA	
		328	no treatment	1300	785	NA	NA	NA	NA	NA	NA	NA	NA	NA	NA	NA	NA	NA	NA	NA	
Patient 3	52	10	7	20	731	NA	NA	+	-	NA	+	-	+	-	-	-	-	-	+	-	V
		46	43	20	902	+	+	NA	NA	NA	ID	-	-	-	-	-	-	+	+	-	
		165	162	20	1011	-	+	+	NA	NA	+	-	+	-	-	+	-	+	+	-	
		335	332	20	884	-	+	+	NA	NA	-	-	-	-	-	-	-	-	-	-	
Patient 4	39	18	no treatment	100000	794	+	+	NA	-	NA	+	-	+	-	-	+	-	+	+	-	V
		42	no treatment	12000	653	NA	NA	NA	NA	NA	+	+	+	+	+	+	+	+	+	-	
		161	no treatment	2500	595	NA	NA	NA	NA	NA	NA	NA	NA	NA	NA	NA	NA	NA	NA	NA	
		332	no treatment	1600	755	NA	NA	NA	NA	NA	NA	NA	NA	NA	NA	NA	NA	NA	NA	NA	

Acute HIV-1 infection stages definitions [1]:
Stage I. vRNA+ (PCR), all other tests negative
Stage II. Above plus p24+ (ELISA), all other tests negative
Stage III. Above plus HIV-1-specific-antibody+ (ELISA)
Stage IV. Above plus HIV-1-specific-antibody+/- (western blot); p31-
Stage V. Above plus HIV-1-specific-antibody+ (western blot); p31-
Stage VI. Above plus HIV-1-specific-antibody+ (western blot); p31+
[a]Uni-Gold™ Recombigen® HIV-1/2 immunoassay (Trinity Biotech); [b]Determine™ HIV-1/2 Ag/Ab test (Alere); [c]p24-antigen test (Roche); [d]Centaur® HIV Ag/Ab Combo (ADVIA)
Abbreviations. ID: indeterminate; NA: not available.

Supplementary Table 2. Proviral sequences detected in patients with treatment initiation during chronic HIV-1 infection.

Patient ID	Manuscript ID	Days on therapy	Viral load at therapy start (copies/mL)	CD4 count at therapy start (cells/mm^3)	Duration of uncontrolled viremia Pre-therapy	Cells Assayed	Number of intact sequences	Number of defective sequences*	Proportion of intact sequences	Intact sequences/million PBMC	Total HIV-sequences/million PBMC
382383	Pt 5	18	Unknown	311	>4yr 3mo	3580000	1	174	1%	0.3	48.9
201476	Pt 6	70	130,000	395	>7yr 3mo	1630000	3	61	5%	1.8	39.3
529440	Pt 7	182	32,895	153	>12yr 10mo	1310000	1	46	2%	0.8	35.9

*Only gel bands over the size of ~6000 base pairs were sequenced; all shorter and specific gel bands were counted as defective viral genomes with large deletion(s).

Supplementary Table 3. Proviral sequences detected in patients with treatment initiation during acute HIV-1 infection.

Patient ID	Days post last PCR negative test	Days post first PCR positive test	Days post treatment initiation	Viral Load (copies/mL)	CD4 (cells/ mm^3)	Treatment	HIV DNA genome classification					Total HIV sequences detected	Number of cells sampled	Intact sequences/million PBMC	Intact + Defective HIV-sequences/million PBMC	ddPCR total HIV DNA/million PBMC
							Intact	Psi-defect	Premature Stop Codon	Hypermutated	Large deletions					
Patient 1	5	2	0	3900	851	TDF,FTC,EFV,RAL	9	0	1	0	1	11	4107600	2.2	3	15
		42	40	20	667	TDF,FTC,EFV,RAL	3	0	1	0	3	7	418950	7.2	17	201
		164	162	20	658	TDF,FTC,EFV,RAL	1	0	0	0	0	1	1373400	0.7	1	76
Patient 2	10	7	NA	380000	457	no treatment	1	0	0	0	0	1	1256850	0.8	1	33
		42	NA	37000	596	no treatment	4	0	1	5	5	15	2116800	1.9	7	60
		164	NA	2600	448	no treatment	2	0	0	2	1	5	1474200	1.4	3	28
		328	NA	1300	785	no treatment	1	0	0	3	2	6	1921500	0.5	3	55
Patient 3	52	10	7	20	731	TDF,FTC,EFV	6	4	0	2	29	41	1776000	3.4	23	480
		46	43	20	902	TDF,FTC,EFV	0	0	2	0	12	14	705717	<1.3	20	1369
		165	162	20	1011	TDF,FTC,EFV	0	1	0	0	1	2	904800	<1	2	106
		335	332	20	884	TDF,FTC,EFV	NA	NA	NA	NA	NA	NA	NA	NA	NA	8
Patient 4	39	18	NA	100000	794	no treatment	26	0	2	3	43	74	835553	31.1	89	2379
		42	NA	12000	653	no treatment	25	1	5	2	41	74	1319760	18.9	56	997
		161	NA	2500	595	no treatment	2	0	0	0	1	3	414000	4.8	7	319
		332	NA	1600	755	no treatment	7	3	1	4	23	38	1845367	3.8	21	150

Abbreviations: TDF tenofavir, FTC emtricitabine, EFV efavirenz, RAL raltegravir

Supplementary Table 4. Frequency of single-base substitution mutations in HIV-1 DNA sequences.

Patient ID	Days post last PCR negative test	Days post first PCR positive test	Viral Load (copies/mL)	CD4 (cells/mm^3)	Treatment	Number of cells sampled	Intact HIV Count	Base substitutions count minimum	Base substitutions count maximum	Base substitutions count median
Pt 1	5	2	3900	851	TDF, FTC, EFV, RAL	4107600	9	1	6	3
		42	20	667	TDF, FTC, EFV, RAL	418950	3	5	7	6
		164	20	658	TDF, FTC, EFV, RAL	1373400	1	NA	NA	NA
Pt 2	10	7	380000	457	no treatment	1256850	1	NA	NA	NA
		42	37000	596	no treatment	2116800	4	4	10	8
		164	2600	448	no treatment	1474200	2	18	18	18
		328	1300	785	no treatment	1921500	1	NA	NA	NA
Pt 3	52	10	20	731	TDF, FTC, EFV	1776000	6	2	4	3
		46	20	902	TDF, FTC, EFV	705717	0	NA	NA	NA
		165	20	1011	TDF, FTC, EFV	904800	0	NA	NA	NA
		335	20	884	TDF, FTC, EFV	NA	NA	NA	NA	NA
Pt 4	39	18	100000	794	no treatment	835553	26	0	21	4
		42	12000	653	no treatment	1319760	25	0	17	5
		161	2500	595	no treatment	414000	2	11	11	11
		332	1600	755	no treatment	1845367	7	15	66	39

Supplementary Table 5. Longitudinal changes in the prevalence of sequence variants in defined, HLA-B5801-restricted CTL epitopes in study subject 4.

Days-Post-Detection	Total Intact Genomes Sampled	Nef82-91: KF9 and AL9[1]		Nef127-135: YY9[2]		Nef135-143: YF9[3]	
		KAAFDLSFFL	KGAFDLSFFL	YTPGPGVRY	YTPGPGVRF	YPLTFGWCF	FPLTFGWCF
18	26	100%	0%	100%	0%	100%	0%
42	25	100%	0%	100%	0%	100%	0%
161	2	50%	50%	100%	0%	100%	0%
332	7	29%	71%	57%	43%	57%	43%

[1]Nef82-90 contains overlapping epitopes KF9 (Nef82-90: KAALDLSHF) and AL9 (Nef83-91: AAVDLSHFL); KgAFDLSFFL is a known escape-epitope of KAAFDLSFFL [2,3]

[2]Nef127-135: YY9; YTPGPGVRf has not been observed in patients, but YTPGPGVRY is known to be targeted by CTL via EliSpot [3,4]

[3]Nef135-143: YF9; fPLTFGWCF is a known variant of YPLTFGWCF [3,5]

Reference:

1. Fiebig, E. W. *et al.* Dynamics of HIV viremia and antibody seroconversion in plasma donors: implications for diagnosis and staging of primary HIV infection. *AIDS* **17**, 1871–9 (2003).
2. Leslie, A. *et al.* Transmission and accumulation of CTL escape variants drive negative associations between HIV polymorphisms and HLA. *J. Exp. Med.* **201**, 891–902 (2005).
3. Los Alamos HIV Molecular Immunology Database. Los Alamos CTL/CD8+ T-Cell Epitope Database. (2018).
4. Chopera, D. R. *et al.* Virological and Immunological Factors Associated with HIV-1 Differential Disease Progression in HLA-B*58:01-Positive Individuals. *J. Virol.* **85**, 7070–7080 (2011).
5. Navis, M. *et al.* A Nonprogressive Clinical Course in HIV-Infected Individuals Expressing Human Leukocyte Antigen B57/5801 Is Associated with Preserved CD8 $^{+}$ T Lymphocyte Responsiveness to the HW9 Epitope in Nef. *J. Infect. Dis.* **197**, 871–879 (2008).
