# Supplementary material for: HIV-1 DNA sequence diversity and evolution during acute subtype C infection
Source: Nat Commun. 2019 Jun 21;10:2737. doi: 10.1038/s41467-019-10659-2 (PMC6588551; doi:10.1038/s41467-019-10659-2)
Supplement: Supplementary file 3 — Reporting Summary [file 41467_2019_10659_MOESM3_ESM.pdf]

nature research

Corresponding author(s): Mathias Lichterfeld

Last updated by author(s): Mar 17, 2019

# Reporting Summary

Nature Research wishes to improve the reproducibility of the work that we publish. This form provides structure for consistency and transparency in reporting. For further information on Nature Research policies, see Authors & Referees and the Editorial Policy Checklist.

## Statistics

For all statistical analyses, confirm that the following items are present in the figure legend, table legend, main text, or Methods section.

| n/a | Confirmed | |
|---|---|---|
| ☐ | ☒ | The exact sample size (*n*) for each experimental group/condition, given as a discrete number and unit of measurement |
| ☒ | ☐ | A statement on whether measurements were taken from distinct samples or whether the same sample was measured repeatedly |
| ☐ | ☒ | The statistical test(s) used AND whether they are one- or two-sided<br>*Only common tests should be described solely by name; describe more complex techniques in the Methods section.* |
| ☒ | ☐ | A description of all covariates tested |
| ☒ | ☐ | A description of any assumptions or corrections, such as tests of normality and adjustment for multiple comparisons |
| ☒ | ☐ | A full description of the statistical parameters including central tendency (e.g. means) or other basic estimates (e.g. regression coefficient) AND variation (e.g. standard deviation) or associated estimates of uncertainty (e.g. confidence intervals) |
| ☒ | ☐ | For null hypothesis testing, the test statistic (e.g. *F*, *t*, *r*) with confidence intervals, effect sizes, degrees of freedom and *P* value noted<br>*Give P values as exact values whenever suitable.* |
| ☒ | ☐ | For Bayesian analysis, information on the choice of priors and Markov chain Monte Carlo settings |
| ☒ | ☐ | For hierarchical and complex designs, identification of the appropriate level for tests and full reporting of outcomes |
| ☒ | ☐ | Estimates of effect sizes (e.g. Cohen's *d*, Pearson's *r*), indicating how they were calculated |

*Our web collection on statistics for biologists contains articles on many of the points above.*

## Software and code

Policy information about availability of computer code

| | |
|---|---|
| Data collection | No software was used. |
| Data analysis | Custom code HIVSeqinR version 2.6 was used to categorize HIV DNA sequences into "intact" or various types of "defective" viral genomes. We have noted in the manuscript text that the source code is freely available in GitHub. |

For manuscripts utilizing custom algorithms or software that are central to the research but not yet described in published literature, software must be made available to editors/reviewers. We strongly encourage code deposition in a community repository (e.g. GitHub). See the Nature Research guidelines for submitting code & software for further information.

## Data

Policy information about availability of data

All manuscripts must include a data availability statement. This statement should provide the following information, where applicable:

- Accession codes, unique identifiers, or web links for publicly available datasets
- A list of figures that have associated raw data
- A description of any restrictions on data availability

All raw HIV sequence data that support the findings of this study have been deposited in GenBank. The authors declare that all other data supporting the findings of this study are available within the paper and its supplementary information files.

# Field-specific reporting

Please select the one below that is the best fit for your research. If you are not sure, read the appropriate sections before making your selection.

☒ Life sciences ☐ Behavioural & social sciences ☐ Ecological, evolutionary & environmental sciences

For a reference copy of the document with all sections, see nature.com/documents/nr-reporting-summary-flat.pdf

# Life sciences study design

All studies must disclose on these points even when the disclosure is negative.

| | |
|---|---|
| Sample size | Only four patients were sampled due to cost and the rarity of longitudinal samples available from hyperacutely-infected patients. No sample-size calculation was performed. |
| Data exclusions | No data were excluded from the analyses. |
| Replication | Not applicable. |
| Randomization | Patients were non-randomly selected to each represent a unique clinical course based on their baseline acute infection stages and their subsequent treatment status. All four patients (Pt) were selected for having protective human leukocyte antigen (HLA) genotypes (Table 1) to shed light on the dynamics of cytotoxic T cells (CTLs) driven viral evolution over time. |
| Blinding | Not applicable. |

# Reporting for specific materials, systems and methods

We require information from authors about some types of materials, experimental systems and methods used in many studies. Here, indicate whether each material, system or method listed is relevant to your study. If you are not sure if a list item applies to your research, read the appropriate section before selecting a response.

## Materials & experimental systems

| n/a | Involved in the study | |
|---|---|---|
| ☒ | ☐ | Antibodies |
| ☒ | ☐ | Eukaryotic cell lines |
| ☒ | ☐ | Palaeontology |
| ☒ | ☐ | Animals and other organisms |
| ☐ | ☒ | Human research participants |
| ☒ | ☐ | Clinical data |

## Methods

| n/a | Involved in the study | |
|---|---|---|
| ☒ | ☐ | ChIP-seq |
| ☒ | ☐ | Flow cytometry |
| ☒ | ☐ | MRI-based neuroimaging |

## Human research participants

Policy information about studies involving human research participants

| | |
|---|---|
| Population characteristics | The FRESH cohort is an observational, prospective cohort launched in 2012 in Umlazi, Durban, South Africa where high-risk HIV-negative women aged 18-23 were recruited to received twice-weekly HIV-1 RNA PCR testing by finger prick during 96 weeks of surveillance, later revised to 48 weeks. |
| Recruitment | No participants were recruited for this specific study. We used banked PBMC samples. |
| Ethics oversight | This study was approved by the Biomedical Research Ethics Committee of the University of KwaZulu-Natal and the Institutional Review Board of Massachusetts General Hospital. All participants provided written informed consent. |

Note that full information on the approval of the study protocol must also be provided in the manuscript.
